# Unilateral Isolated Hypoglossal Nerve Palsy Secondary to Tonsillitis

**DOI:** 10.7759/cureus.20291

**Published:** 2021-12-09

**Authors:** Ryo Kawaura, Masami Ohnishi

**Affiliations:** 1 Department of Head and Neck Surgery-Otolaryngology, Ogaki Municipal Hospital, Ogaki, JPN

**Keywords:** corticosteroid, tonsillitis, unilateral, isolated, hypoglossal nerve palsy

## Abstract

Hypoglossal nerve palsy is usually associated with glossopharyngeal nerve, vagus nerve, and accessory nerve palsy, and the occurrence of hypoglossal nerve palsy alone is rare. We report a case of a 41-year-old man with unilateral isolated hypoglossal nerve palsy. The patient was aware of a leftward deviation of the tongue along with pharyngeal pain. The pharyngeal pain was quickly relieved by antibiotic treatment, but the deviation of the tongue did not improve, and the patient was referred to our hospital. As a result of the medical examination, a single paralysis of the left hypoglossal nerve and mild swelling of the left lingual tonsil up to the left palatine tonsil were observed. Various tests were performed, but there were no significant abnormal findings other than a suggestion of mild tonsillitis. We diagnosed the patient as idiopathic or tonsillitis-induced unilateral hypoglossal nerve palsy and started medical treatment with corticosteroids and methylcobalamin. The hypoglossal nerve palsy showed a tendency to improve after one month of onset and was almost cured by two months of onset.

## Introduction

The hypoglossal nerve is the 12th cranial nerve and is composed solely of motor nerves. Hypoglossal nerve palsy (HNP), often encountered as a complication of nerve injury in head and neck surgery, may result from tumors, strokes, or neurological diseases. It usually occurs in combination with glossopharyngeal, vagus, and accessory nerve palsy, but the isolated occurrence of HNP is rare [1]. We report a case of unilateral isolated HNP, which could have been secondary to tonsillitis, although the cause could not be identified by various tests. Although no reports of steroid treatment have been confirmed in HNP cases after tonsillitis, HNP was almost cured by corticosteroids treatment in this case.

## Case presentation

A 41-year-old man with a history of hypertension visited our hospital complaining of sore throat and left tongue deviation. He was an office worker and a current smoker with 15 cigarettes a day for 20 years. His height was 179 cm and his weight was 85 kg. He had been aware of a sore throat and a deviation of the tongue to the left side two days before he visited our department. He went to see an otolaryngology practitioner, who diagnosed tonsillitis and started treatment with oral antibiotics (cefditoren-pivoxil). The next day (the day before he visited our department), he still had difficulty swallowing and underwent computed tomography (CT) scan of the head at his local physician's office, but there was no abnormality. Therefore, he was referred to the neurology department of our hospital, and on the same day, he also visited the otolaryngology department for a consult.

**Figure 1:**
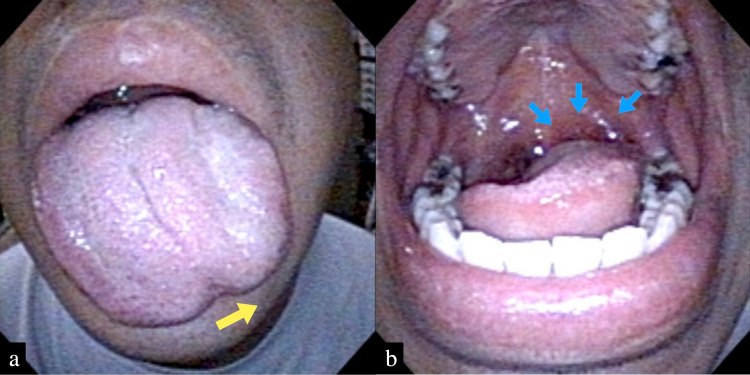
Images of endoscopy When the patient opened his mouth, (a) his tongue deviated to the left without atrophy (yellow arrow) and (b) the left palatine tonsil and left lingual tonsil showing erythema and swelling (blue arrows) (b). There was no abnormal sensation of the tongue and no abnormal taste perception.

On examination by the neurologist, the patient's eye movements were unrestricted, pupils were 3 mm, and light reflexes were prompt bilaterally. There was no difference in facial sensation between the right and left sides, and no facial nerve palsy. Although his tongue was shifted to the left side, there was no difference in tongue sensation between the right and left sides, and no abnormal taste perception. There were also no motor or sensory deficits in his extremities or trunk. On our examination, the left palatine tonsil and left lingual tonsil showed erythema and swelling, as well as mild dysarthria (Figures 1a, 1b). There were no inflammatory findings in the pharyngeal tonsils or larynx. And no cervical lymphadenopathy was noted. 

**Figure 2:**
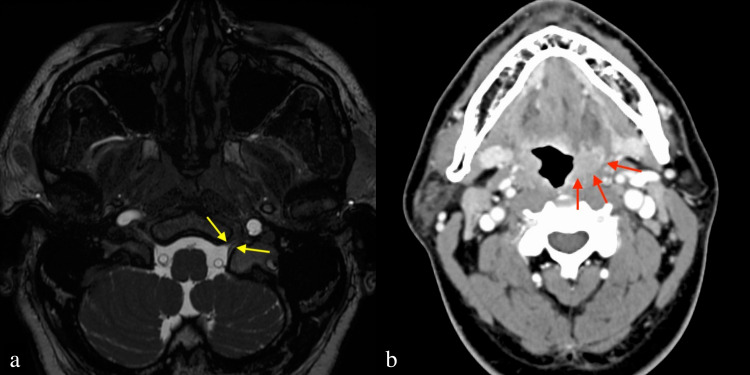
MRI and contrast-enhanced computed tomography (CECT) images at the initial visit (a) MRI image showing no notable lesion found nearby the left hypoglossal canal (yellow arrows). (b) CECT image showing swelling of the left lingual tonsils and palatine tonsils (red arrows), but no neoplastic lesions were found. CISS: constructive interference in steady-state

Blood tests at the initial visit showed mildly elevated liver enzymes and high triglyceride levels, but no other biochemical or electrolyte abnormalities were found. The white blood cell count was increased to 10,240/μL, while the C-reactive protein (CRP) was within the reference range at 0.22 mg/dL. The patient had already been treated with antibiotics for tonsillitis, suggesting that the tonsillitis was in the process of healing or was minor tonsillitis. Furthermore, tumor markers were not abnormal and viral tests showed the previous infection (Table 1). Imaging studies included head magnetic resonance imaging (MRI), magnetic resonance angiography, cervical contrast-enhanced CT, and cervical ultrasonography, all of which showed no abnormalities such as intracranial lesions, cerebrovascular abnormalities, or neck tumors (Figures 2a, 2b).

**Table 1:** Results of the blood test at the initial visit The values given inside brackets indicate the normal value in our department. T-Bil: total bilirubin; AST: aspartate aminotransferase; ALT: alanine aminotransferase; LDH: lactate dehydrogenase; γ-GTP: gamma-glutamyltransferase; ALP: alkaline phosphatase; TP: total protein; CPK: creatine phosphokinase; BUN: blood urea nitrogen; LDL-C: low-density lipoprotein cholesterol; HDL-C: high-density lipoprotein cholesterol; HbA1c: hemoglobin A1c; CRP: C-reactive protein; WBCs: white blood cells; RBCs: red blood cells; fT3: free tri-iodothyronine; fT4: free thyroxine; TSH: thyroid-stimulating hormone; CEA: carcinoembryonic antigen; CA19-9: carbohydrate antigen 19-9; SCC: squamous cell carcinoma antigen; sIL-2R: soluble interleukin-2 receptor; EBV VCA: Epstein-Barr virus viral capsid antigen; EBNA: Epstein-Barr virus nuclear antigen; HSV: herpes simplex virus; VZV: varicella-zoster virus; IgM: immunoglobulin M; IgG: immunoglobulin G

Blood test	Value	Unit	Reference range
Biochemistry			
T-Bil	1.3	mg/dL	0.2-1.2
AST	26	IU/L	5-40
ALT	53	IU/L	3-35
LDH	174	IU/L	124-222
γ-GTP	80	IU/L	<56
ALP	299	IU/L	104-338
TP	7.6	g/dL	6.5-8.2
Albumin	5.0	g/dL	3.5-5.0
CPK	97	IU/L	35-200
BUN	12.3	mg/dL	8.0-23.0
Creatinine	0.94	mg/dL	0.62-1.10
LDL-C	135	mg/dL	<140
HDL-C	42	mg/dL	40-70
Triglyceride	323	mg/dL	50-149
Sodium	140	mEq/L	136-148
Potassium	4.1	mEq/L	3.6-5.0
Chloride	104	mEq/L	99-113
Calcium	9.7	mg/dL	8.0-11.0
Glucose	97	mg/dL	70-110
HbA1c	5.3	%	4.6-6.2
CRP	0.22	mg/dL	<0.25
Peripheral blood			
WBCs	10240	/μL	3500-9900
RBCs	617	×10^4^/μL	395-540
Hemoglobin	17.6	g/dL	12.7-16.4
Hematocrit	51.8	%	37.8-48.2
Platelets	26	×10^4^/μL	12-40
Thyroid hormone			
fT3	3.45	pg/mL	2.3-4.1
fT4	1.02	ng/dL	0.88-1.50
TSH	1.703	μIU/mL	0.4-4.2
fT3	3.45	pg/mL	2.3-4.1
Tumor maker			
CEA	2.1	ng/mL	<5.0
CA19-9	6.7	U/mL	<37
SCC	<1	ng/mL	<1.5
sIL-2R	412	U/mL	122-496
Pathogen-specific antibodies/antigens			
EBV VCA-IgM	0.0	Times	<0.5
EBV VCA-IgG	9.3	Times	<0.5
EBNA-IgG	1.9	Times	<0.5
HSV-IgM	0.19	Times	<0.80
HSV-IgG	41.3	Times	<2.0
VZV-IgM	0.09	Times	<0.85
VZV-IgG	51.9	Times	<6.0
EBV VCA-IgM	0.0	Times	<0.5

The patient's clinical symptom was a single paralysis of the left hypoglossal nerve. In addition, only slight redness and swelling of the left palatine tonsil and lingual tonsil due to tonsillitis were observed; the symptoms of pharyngeal pain tended to improve. At this point, we made a diagnosis of unilateral HNP caused by idiopathic or tonsillitis. We started medical treatment with oral prednisolone tapering for nine days (30 mg for three days, 20 mg for three days, and 10 mg for three days) and methylcobalamin, following the treatment of peripheral facial paralysis. The paralysis showed a tendency to improve after one month of onset and was almost completely healed by two months of onset. Due to the patient’s wishes, our examinations ended at this time.

## Discussion

The hypoglossal nerve innervates the muscles that control tongue movement in all tongue muscles except the vagally innervated palatoglossus muscle, which is closely related to tongue movement necessary for mastication, swallowing, and speech production [2]. HNP is caused by a variety of diseases in the region from the hypoglossal nucleus through the hypoglossal canal to the periphery; paralysis of the genioglossus muscle, which has the effect of protruding the tongue, causes tongue shift to the affected side. In addition to impairing pronunciation and swallowing, prolonged paralysis leads to tongue atrophy and fasciculation on the affected side [3]. Usually, unilateral HNP is combined with paralysis of the glossopharyngeal, vagus, and accessory nerves and appears as the last four cranial nerves; HNP is rarely seen alone [1].

According to Keane, among 100 cases of HNP, the tumor was the most common cause (49 cases), followed by trauma (12 cases), stroke (six cases), hysteria (six cases), multiple sclerosis (six cases), surgery (five cases), Guillain-Barre syndrome (four cases), and infection (four cases) [1]. In our case, tonsillitis before the onset of HNP was not severe, but the patient had no history of surgery, trauma, or hysteria, and no imaging tests showed stroke and neurological disorders; his HNP was considered to be idiopathic or secondary to tonsillitis. As for paralysis of the hypoglossal nerve due to infection, there are some reports of paralysis associated with infectious mononucleosis caused by the Epstein-Barr virus [4-7] and neuritis caused by influenza virus [8]. In addition, there have been a few reports of HNP after bacterial tonsillitis; Kovalev and Clarenbach pointed out the possibility of the abnormal running of the hypoglossal nerve in his report [9], while Sakemi et al. stated that inflammation of the periphery of the anterior wall of the hypopharynx due to lingual tonsillitis can anatomically cause inflammatory spillover to the hypoglossal nerve [10]. There is a report that the prognosis of HNP associated with infection recovers within six months [11], while Sakemi et al. reported that HNP and atrophy of the tongue, secondary to prolonged lingual tonsillitis, remained for more than seven years [10]. Further reports will be needed, but from the perspective of the spread of inflammation to nerves, it is expected that the stronger and longer the underlying inflammation, the more irreversible the effects on nerves.

In this case, we treated the patient with oral prednisolone and methylcobalamin according to the treatment of peripheral facial nerve palsy. If this case is HNP due to tonsillitis, not idiopathic, this is the first report of prednisolone treatment with HNP after tonsillitis as far as we know. Mori et al. treated the patient with oral prednisolone and methylcobalamin in a case of idiopathic unilateral HNP, and obtained an improvement of the palsy; in this report, they suggest the efficacy of administering corticosteroids and methylcobalamin in the early stage of HNP [12]. In a meta-analysis of the facial nerve palsy, Ramsay et al. reported that corticosteroid administration within one week of onset increased the remission rate by 17% [13]. Even in the case of idiopathic or secondary to infection HNP, early corticosteroid administration seems supportive, given that neuroedema is the primary pathology. According to Donen et al., recovery of HNP can be achieved without any special treatment [11]. In our case, the HNP was secondary to relatively minor tonsillitis, and the palsy might have recovered spontaneously without the aforementioned medication. However, it may be difficult to determine whether the HNP is truly caused by infection or is idiopathic; if oral corticosteroids can be administered early in the course of the palsy, we should be actively considered.

## Conclusions

We experienced a case of unilateral isolated HNP in which the cause could not be identified by various tests. Based on the clinical course, it was considered to be idiopathic or tonsillitis secondary HNP. Since prolonged HNP can lead to tongue atrophy and residual tongue deviation, unilateral isolated HNP should be considered for treatment with corticosteroids as soon as possible.

## References

[1] Keane JR (1996). Twelfth-nerve palsy. Analysis of 100 cases. Arch Neurol.

[2] Asada Y, Matsuura K (2014). Zekkashinkei (hypoglossal nerve). JOHNS.

[3] Oda T, Abe H, Nakagawa S (1979). Internal Medicine Seminar PN1 Neurological Diagnosis. https://www.molcom.jp/products/detail/118102/.

[4] Sibert JR (1972). Hypoglossal nerve palsy complicating a case of infectious mononucleosis. Postgrad Med J.

[5] Wright GD, Lee KD (1980). An isolated right hypoglossal nerve palsy in association with infectious mononucleosis. Postgrad Med J.

[6] Zafeiriou DI, Pavlou E (2004). Images in clinical medicine: hypoglossal nerve palsy. N Engl J Med.

[7] van Baalen A, Petersen B, Stephani U (2006). Infectious mononucleosis and unilateral tongue writhing. Neurology.

[8] Felix JK, Schwartz RH, Myers GJ (1976). Isolated hypoglossal nerve paralysis following influenza vaccination. Am J Dis Child.

[9] Kovalev D, Clarenbach P (2000). Isolated reversible unilateral paresis of hypoglossal nerve in tonsillitis--case report. [Article in German]. Nervenarzt.

[10] Sakemi H, Taniguchi H, Iseki T (2002). Zetsuhentouen ni zokuhatsu-shita masshousei zekkashinkeishougai ni-yoru hansoku zetsuishuku. [Article in Japanese]. JIM.

[11] Donen M, Oda M, Saito S, Kobayashi I, Totsuka Y (2001). A case of unilateral tongue atrophy caused by idiopathic hypoglossal nerve palsy. J Jpn Soc Oral Surg.

[12] Mori K, Fujiwara K, Shimada J (2015). A case of idiopathic [sic] unilateral hypoglossal nerve palsy. J Jpn Oral Diag Soc.

[13] Ramsey MJ, DerSimonian R, Holtel MR, Burgess LP (2000). Corticosteroid treatment for idiopathic facial nerve paralysis: a meta-analysis. Laryngoscope.

